# Twenty years of anastomotic stenosis combined with tracheocolonic fistula after colon replacement esophagectomy: a case report

**DOI:** 10.3389/fonc.2024.1471603

**Published:** 2024-12-12

**Authors:** Xiaofang Li, Liutao Zou, Lifeng Shi, Xueting Zheng, Cuifang Xu, Jichao Guo

**Affiliations:** ^1^ Department of Respiratory Medicine, The First Affiliated Hospital/College of Clinical Medicine of Henan University of Science and Technology, Luoyang, China; ^2^ Department of Thoracic Surgery, Guangxi Chest Hospital, Liuzhou, China; ^3^ Department of Thoracic Surgery, Shandong Provincial Hospital Affiliated to Shandong First Medical University, Jinan, China; ^4^ Department of Gastroenterology, Shulan (Ji Nan) Hospital, Jinan, China; ^5^ Lanshan District People’s Hospital, Department of Thoracic Surgery, Linyi, Shandong, China

**Keywords:** tracheoesophageal fistula, esophageal stenosis, esophageal stent, tracheal stent, case report

## Abstract

Esophageal stricture is the most common and disabling complication of esophageal injury caused by ingestion of corrosive substances. In our case, the patient developed esophageal stenosis due to ingestion of strong alkaline substances and underwent colon replacement surgery after repeated failed dilation treatments. After surgery, anastomotic stenosis and tracheocolonic fistula occurred successively, and the entire diagnosis and treatment cycle of this disease lasted for more than 20 years. Based on experience and the actual situation of the patient, we conclude that esophageal stents should be the primary treatment option, while tracheal stents should be carefully selected, and secondary surgery is not recommended.

## Background

Esophageal injury caused by ingestion of corrosive substances is a relatively rare but potentially destructive event, which can lead to death in severe cases. Esophageal stricture caused by ingestion of corrosive substances is the most common and disabling long-term complication, typically occurring within 4 months after ingestion (1). Endoscopic dilation is the first-line treatment for early esophageal stenosis (2, 4), and esophageal reconstruction should be considered after five to seven unsuccessful attempts at dilation (5).

## Case presentation

A 52-year-old man, 21 years ago, accidentally ingested industrial alkali (mainly sodium hydroxide) in an amount of approximately 40 mL. After active treatment such as gastric lavage in the local hospital’s gastroenterology department, multiple gastroscopy examinations showed persistent fibrotic stenosis in the esophagus. Over the next 2 years, multiple balloon dilations were performed under gastroscopy, but the effect was not satisfactory. Later, stage gastroscopy cannot pass through the esophagus smoothly; therefore, esophageal replacement surgery was recommended. The patient underwent total esophagectomy colon replacement surgery in 2005. Dysphagia occurred after oral eating for a period of time, and gastroscopy revealed two anastomotic strictures 23 cm and 31 cm away from the incisor. Within 3 years, the anastomotic cicatrix contracture and stenosis worsened progressively, and finally, the gastric tube placement under the guidance of gastroscopy failed. After many hospitals said that there was no special treatment, the patient gave up treatment. Under the guidance of a doctor, the patient underwent esophageal dilation on their own by a plastic tube after discharge. The inner diameter of the tube gradually increased and later reached 13 mm, allowing for normal oral feeding. This condition was maintained for 15 years, and the patient had a good quality of life. One year ago, the patient went to the hospital for a follow-up examination and underwent a bronchoscopy. Three locations were found to have tracheoesophageal fistulas, with the largest fistula opening being approximately 1 cm. Subsequently, a tracheal metal-covered stent was placed. After implantation, the patient repeatedly developed cough and sputum and pulmonary infection and came to our hospital for further treatment.

**Figure 1:**
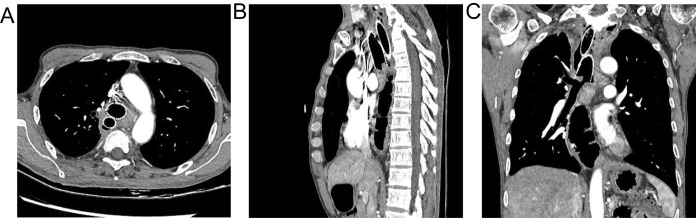
**(A)** the relationship between double stents and fistula was observed from the horizontal view. **(B)** observed from sagittal a double support with fistula. **(C)** the adjacent relationship between the double stent and the fistula was observed from the coronal view.

**Figure 2:**
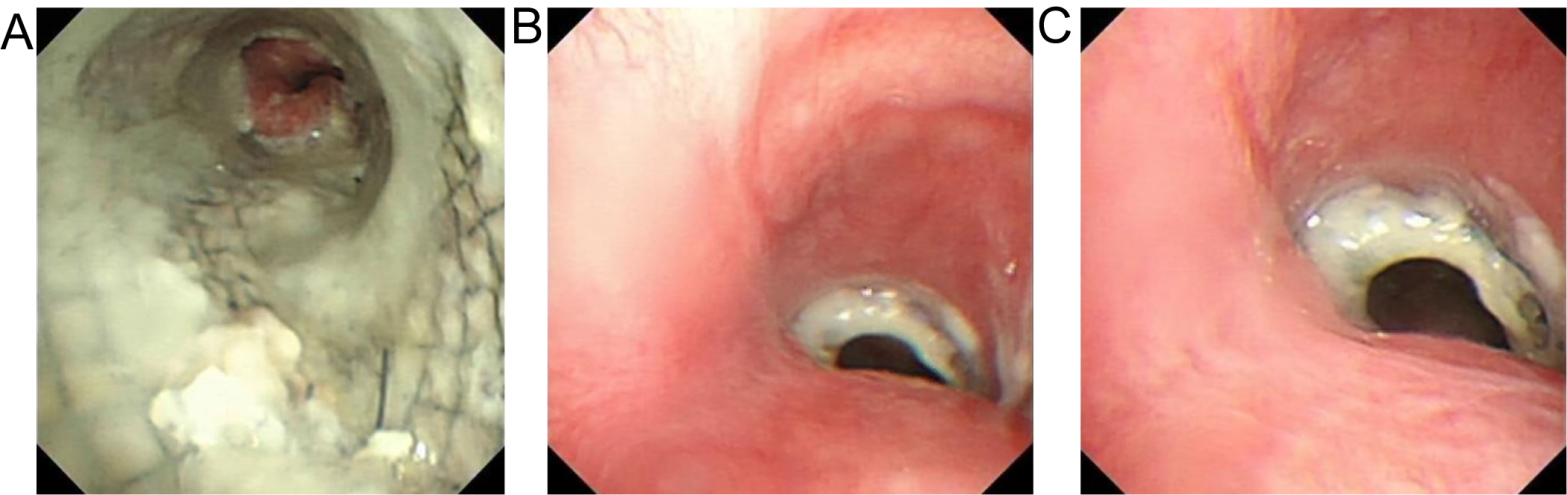
**(A)** the microscopic observation of tracheal stent. **(B)** the microscopic observation of tracheal stent top close shot **(C)** the microscopic observation of tracheal stent top vision.

Chest CT findings are shown in **Figure 1** and **Supplementary Video 1**. The tracheoscopic manifestations are shown in **Figure 2**. We created a schedule of major events during the course of the disease (**Table 1**). Combined with the medical history and current situation, the patient was discharged from the hospital considering that there was no chance of reoperation.

**Table 1:** Major clinical event schedule.

Date	Event	Treatment
2003	Esophageal stricture after ingestion of industrial alkali	Multiple balloon dilations after gastric lavage
2005	Severe esophageal stenosis	Whole tube resection of colon instead of esophagus
6 months after surgery	Anastomotic stenosis	Intermittent balloon dilation for 3 years
2008	Severe anastomotic stenosis	Enteral nutrition was performed by placing a plastic tube into the stomach through the mouth
2023	Follow-up found tracheoesophageal fistula	Tracheal metal stent implantation

## Discussion

In our case, the patient had a narrowing of the esophagus due to ingestion of a strongly alkaline substance. After several failed dilation treatments, colon replacement esophageal surgery was performed. Anastomotic stenosis and esophagotracheal fistula appeared after the operation. The diagnosis and treatment of the whole disease lasted for more than 20 years, and the process was complicated and tortuous. Cases are very rare. Unfortunately, due to the length of time, previous medical records are not currently available. The patient is currently in the state of tracheal stent implantation, and the coverage of the tracheal fistula is incomplete. The self-made plastic nutrition tube is placed into the stomach through the mouth, resulting in poor nutritional status and low quality of life due to repeated lung infections and cough. Based on this patient, we put forward our own treatment opinion.

We prefer comprehensive treatment with esophageal therapy as the main treatment. The main consideration is that from the perspective of nutrition, combined with the patient’s self-placement of self-made nutrition tubes in the past 15 years, we consider that the modified esophageal silicone stent can be placed, which can improve the patient’s nutritional status through oral feeding and is conducive to the healing of the fistula. Meanwhile, the modified esophageal silicone stent can be placed for a long time, the local irritation symptoms are mild, and the patient can operate it by himself without affecting work and life. In addition, the implantation of esophageal stents can seal the fistula opening, fundamentally preventing gastric contents from overflowing into the airway (8).

We do not recommend tracheal stent placement, tracheal esophageal double stent placement, or surgical treatment. Consider the following points: 1) from the perspective of clinical symptoms, the patient’s recurrent cough and sputum were mainly caused by lung and bronchial inflammation caused by gastric contents regurgitating into the trachea, as well as airway irritation caused by stent. However, for tracheoesophageal fistula caused by benign lesions, the placement of an airway stent was only suitable for the treatment during the transitional period of surgery (7), and long-term retention was not appropriate. 2) Dual stent placement for the trachea and esophagus can seal bilateral fistulas, but there will be shear effects between the stents (3, 7), resulting in progressive enlargement of the fistula. In addition, the progressive enlargement of the fistula after the patient’s self-made nutrition tube is placed through the mouth, and the airway stent is placed, further verifying that the placement of double stents is not appropriate. 3) Once benign tracheoesophageal fistula is found, surgical treatment is often considered the key to successful treatment (6). Posterior mediastinal esophagectomy with gastric transposition has a good prognosis in children with esophageal stenosis caused by caustic substances (9, 10). Secondary gastro-esophagectomy was also considered, but the surrounding tissue adhesion and fistula area were large after colon and esophageal replacement surgery. Exposure to local anatomic relationships is very difficult, and secondary replacement surgery and flap implantation are not possible. At the same time, surgical treatment may damage the recurrent laryngeal nerve and peripheral blood vessels, resulting in later dyspnea and a high likelihood of recurrent avascular necrosis, so there is no indication of surgery.

## Conclusion

Based on the above aspects, for patients with tracheocolonic fistula or tracheoesophageal fistula, we proposed a new concept focusing on esophageal treatment: the modified esophageal silicone stent was placed, and the stent could be removed because the implantation of the tracheal stent was only used as a short-term alternative treatment, while the treatment of pulmonary infection was also given. The tracheoscopy was periodically reviewed to observe the changes in the fistula, and growth factors were tried to stimulate the local mucosal tissue proliferation to narrow the fistula so that the long-term coexistence of the fistula and the patient could be achieved even if the fistula could not heal.

## Data Availability

The original contributions presented in the study are included in the article/**Supplementary Material**. Further inquiries can be directed to the corresponding authors.
